# Applicative Characteristics of a New Zirconia Bracket with Multiple Slots

**DOI:** 10.1155/2016/4348325

**Published:** 2016-04-24

**Authors:** Koutaro Maki, Katsuyoshi Futaki, Satoru Tanabe, Mariko Takahashi, Yuta Ichikawa, Tetsutaro Yamaguchi

**Affiliations:** Department of Orthodontics, School of Dentistry, Showa University, 2-1-1 Kitasenzoku, Ohta-Ku, Tokyo 145-8515, Japan

## Abstract

We have developed a new orthodontic bracket with three slots with lubricative properties on the working surfaces and proposed a new orthodontic treatment system employing 0.012−0.014-inch Ni-Ti arch wires. We recruited 54 patients, of which 27 received treatment with the new zirconia bracket with multiple slots system (M group), and the others received treatment with standard edge-wise appliances (control group [C group]). We compared the (1) tooth movement rate at the early stage of leveling; (2) changes in the dental arch morphology before and after leveling; and (3) pain caused by orthodontic treatment. Student's *t*-test was used in all assessments. The tooth movement rate in the maxillomandibular dentition was higher in the M group. The basal arch width, anterior length, and the intercanine width in the maxillary dentition were not significantly different in the two groups; however, the intercanine width in the mandibular dentition was higher in the C group. In assessments of treatment-related pain, the visual analogue pain score was 56.0 mm and 22.6 mm in the C and M groups, respectively. A new zirconia bracket with multiple slots system provided better outcomes with respect to tooth movement rate, treatment period, and postoperative pain, thus indicating its effectiveness over conventional orthodontic systems.

## 1. Introduction

Orthodontic treatment involves the use of wires to apply loads for tooth movement. It has been reported that when the orthodontic force is weak and long-lasting, teeth move quickly with less pain [1]. In this study, we developed a new orthodontic bracket and proposed a new orthodontic treatment system, in which 0.012−0.014-inch Ni-Ti arch wires are used.

We produced an orthodontic bracket with three slots, using a zirconia firing working method that can add lubricative properties to the internal surface of the wire slots and external surface of the brackets. A new zirconia bracket with multiple slots was developed to shorten orthodontic treatment time, relieve pain and discomfort, reduce the restricted function of muscles around the oral cavity, and promote cleansability and esthetics.

The purpose of the present study was to compare this new orthodontic treatment system with conventional treatment systems to clarify differences in the tooth movement rate, clarify changes in the morphology of the dental arch at the early stage of leveling, and assess the degree of pain caused by orthodontic treatment.

## 2. Methods

### 2.1. Bracket Design (Figure 1)

#### 2.1.1. Profile

**Figure 1:**
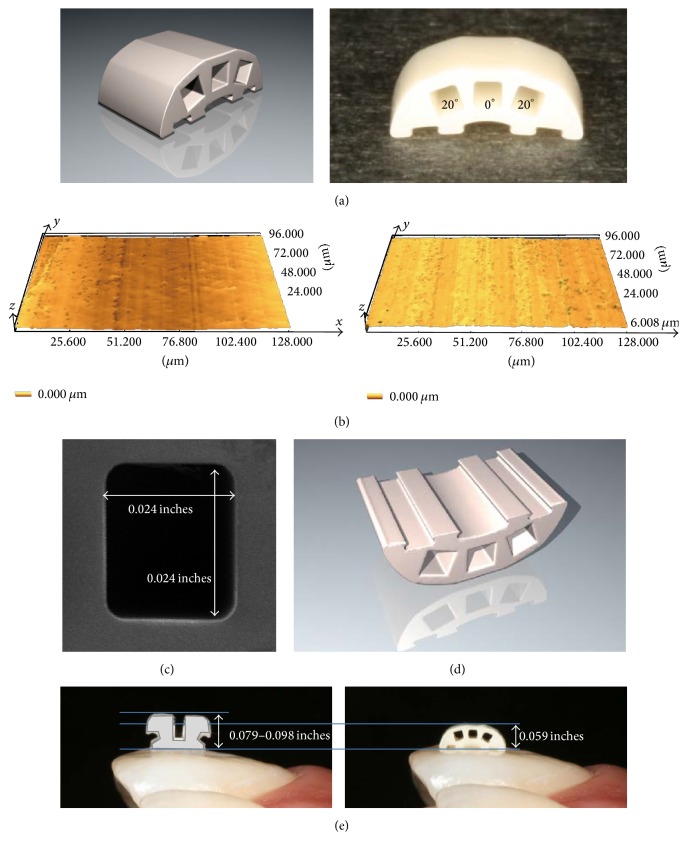
(a) Spherical structure of the bracket surface and slot torque; (b) bracket surface (left) and internal surface (right) of slots (laser microscope, magnification x100) (GC ORTHOLY CORPORATION, Tokyo, Japan); (c) slots (scanning electron microscope, magnification x20); (d) base; and (e) size. Conventional brackets for the anterior tooth area (left) and a new zirconia bracket with multiple slots (right).

The spherical structure of the bracket surface and the slot torque is shown in Figure 1(a). The bracket surface and the internal surface of the slots are smooth due to the zirconia firing working method (Figure 1(b)). Torque is symmetrically applied to both ends of the slot. Therefore, it is unnecessary to change brackets according to tooth type.

#### 2.1.2. Slots (Figure 1(c))

The size of the three square slots is equal.

#### 2.1.3. Base (Figure 1(d))

Strong bond strength can be achieved by a mechanical interlocking force.

#### 2.1.4. Size (Figure 1(e))

Because of the excellent strength of zirconia, a small size and low profile could be used. Furthermore, we succeeded in adding three slots.

## 3. Materials and Methods

### 3.1. Subjects

The subjects were 54 patients (15 males and 39 females) who visited the Department of Orthodontics at the dental hospital attached to Showa University, from whom consent for this research was obtained. This study was performed after receiving approval from the ethics committee at Showa University Dental School (approval number: 2007-28).

Twenty-seven patients (7 males and 20 females aged 11−49 years, mean age: 21 years and 0 months), who received treatment using the new orthodontic treatment system, were classified into a new zirconia bracket with multiple slots group (M group); and 27 patients (8 males and 19 females aged 11−39 years, mean age: 21 years and 8 months), who received treatment using standard edge-wise appliances, were classified into a control group (C group). Regarding the M and C groups, the following three items were compared.

### 3.2. Measurements with Plaster Casts

For plaster casts, hard dental plaster for orthodontic treatment (ORTHO MAX, SHIMOMURA GYPSUM Co., Ltd., Saitama, Japan) was used. Measurement was performed using calipers with divisions of 1/20 mm. The mean of three measurements was calculated. The same clinician performed each measurement.

#### 3.2.1. Tooth Movement Rate at the Early Stage of Leveling

**Figure 2:**
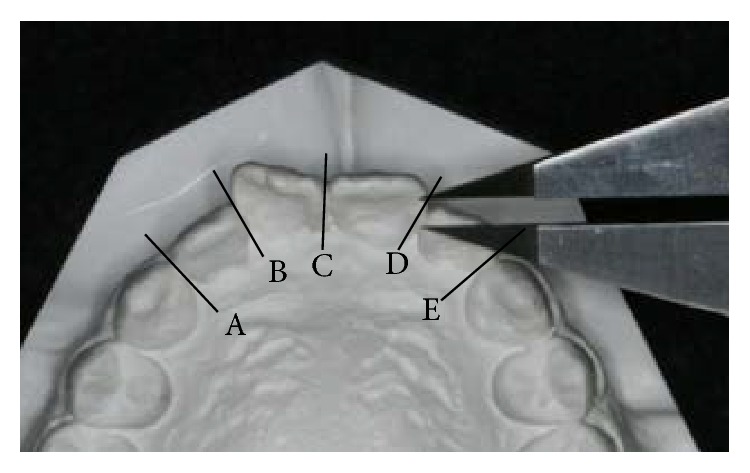
Irregularity index (sum of contact point displacements) (A + B + C + D + E).

The irregularity index before and after leveling was calculated from plaster casts (Figure 2). The completion of leveling was judged using intraoral photographs and plaster casts. A linear functional numerical expressing tooth movement was achieved from the irregularity index before and after leveling and the treatment period required until leveling completion was determined. The gradient of the formula indicates the rate of tooth movement, and when the gradient is large, the rate of tooth movement is high. Regarding the irregularity index after leveling completion, a permissible range was set at a moderate irregularity of 4−6, in reference to the criteria proposed by Little [2].

#### 3.2.2. Changes in the Dental Arch Morphology before and after Leveling

**Figure 3:**
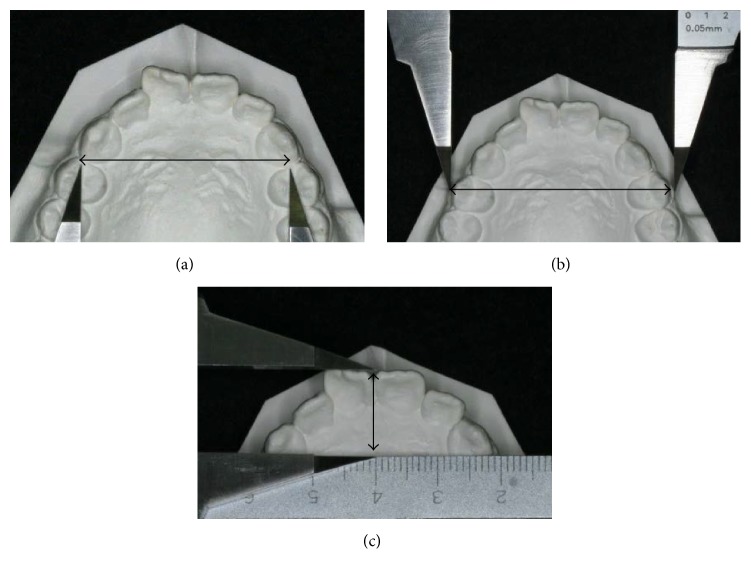
(a) Intercanine width; (b) basal arch width; and (c) anterior length.

We measured the intercanine width (Figure 3(a)), basal arch width (Figure 3(b)), and anterior length (Figure 3(c)) before and after leveling, and a comparison of the extent of change between M and C groups was performed.

#### 3.2.3. Pain due to Orthodontic Treatment

Pain was evaluated based on individual interviews and the visual analogue scale method [3]. A line of 100 mm was drawn on an investigation form, and patients marked the line to show the level of pain they felt during the period of treatment, setting the left end as no pain at all and the right end as unbearable pain. The distance between the left end and the point indicating the level of pain was measured, and a comparison between M and C groups was performed.

### 3.3. Statistical Analysis

Student's *t*-test was used in all cases.

## 4. Results and Discussion

### 4.1. Tooth Movement Rate at the Early Stage of Leveling

**Figure 4:**
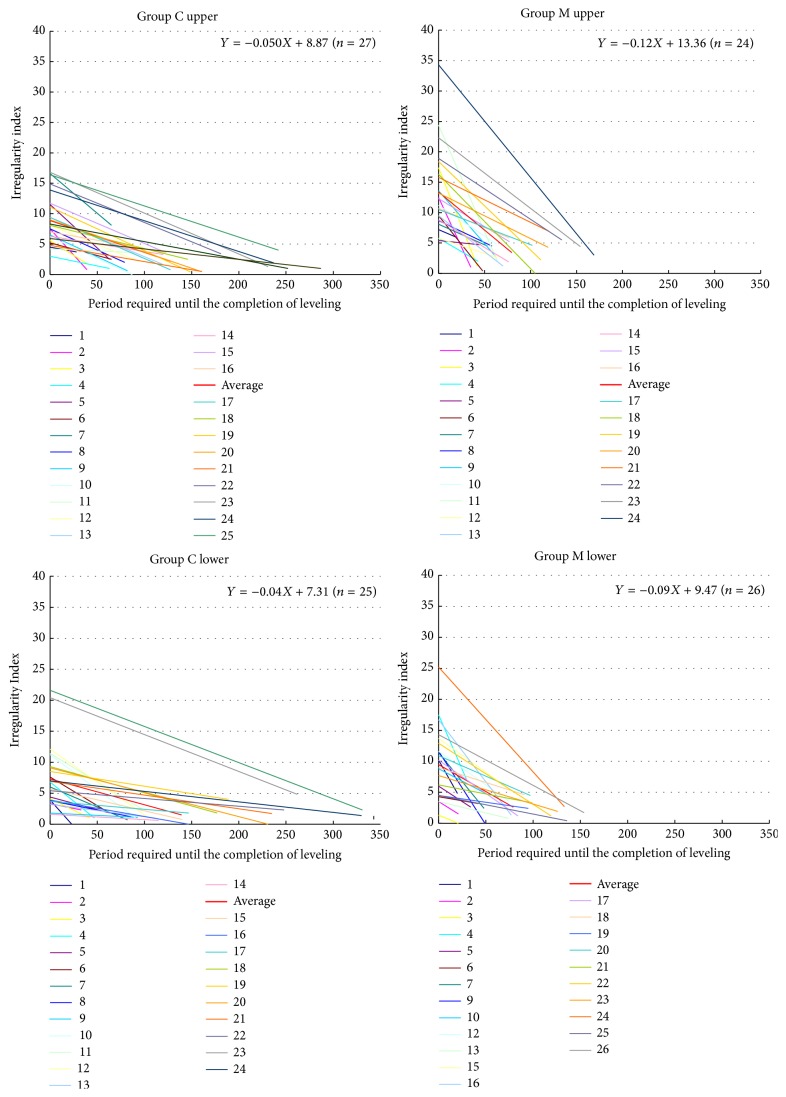
Linear functional formula expressing tooth movement.

The linear functional formula expressing tooth movement is *Y* = −0.05*X* + 8.87 in the C group and *Y* = −0.12*X* + 13.36 in the M group in the maxillary dentition; and *Y* = −0.04*X* + 7.31 in the C group and *Y* = −0.09*X* + 9.47 in the M group in the mandibular dentition (Figure 4). The gradient of the formula indicating the rate of tooth movement was higher in the M group than the C group in the maxillomandibular dentitions.

Furthermore, the mean treatment period was 127.6 days in the C group and 79.7 days in the M group in the maxillary dentition and 139.4 days in the C group and 74.2 days in the M group in the mandibular dentition (Table 1). The tooth movement rate increased in the M group in the maxillomandibular dentitions, in comparison with the C group.

### 4.2. Changes in the Dental Arch Morphology before and after Leveling (Figure 5)

**Figure 6:**
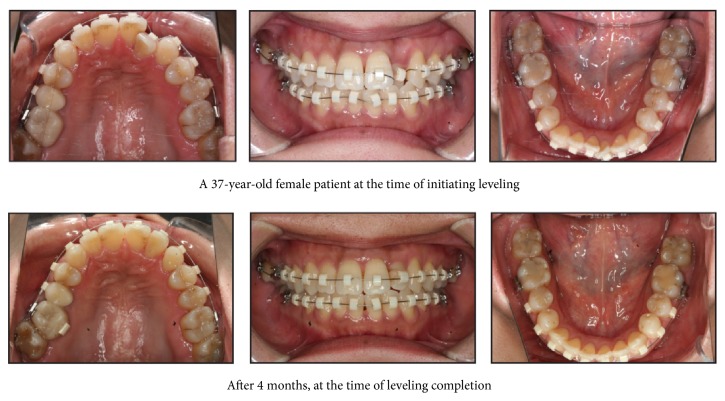
Intraoral photographs before and after leveling completion in the M group.

Regarding the basal arch width and anterior length, no significant differences were noted between the M and C groups. Furthermore, the intercanine width showed no significant difference between the M and C groups in the maxillary dentition; however, in the mandibular dentition, it increased in the C group in comparison with the M group, showing a significant difference (Figure 6).

### 4.3. Pain due to Orthodontic Treatment (Figure 7)

The distance indicating the level of pain was 56.0 mm in the C group and 22.6 mm in the M group. Furthermore, 36.8% of patients in the M group answered that no pain was present, and five of 19 patients reported definite pain. Furthermore, two subjects who had experienced the insertion of conventional orthodontic appliances reported the marked alleviation of discomfort.

The fabrication of veneers, full and partial coverage crowns, fixed partial dentures, posts and/or cores, primary double crowns, implants, implant abutments, and various other dental auxiliary components such as cutting burs, surgical drills, extracoronal attachments, and orthodontic brackets are all employed in the clinical application of zirconia [4]. The biotechnical characteristics of zirconia result in high-quality materials with excellent biocompatibility and aesthetic appearance [5]. Because patients wear orthodontic brackets for months to years, smaller brackets that look similar to natural teeth are preferred for their aesthetic, subtle appearance, which helps patients feel more confident and self-assured while undergoing treatment. Nonmetal brackets offer the attractive combination of aesthetics and performance [6]. Among dental ceramics, zirconia features superior aesthetics and strength [7] and has been applied in the fabrication of orthodontic brackets [8]. We sought to develop brackets that were as small and thin as possible, by using three slots to increase variation in mechanics. In these brackets, the maximum wire size is 0.016−0.016 in for a continuous arch wire and 0.016−0.022 in for a sectional arch wire. Mesiodistal root uprighting and labiolingual torque were achieved with Invisalign® or aligner systems [9].

**Figure 8:**
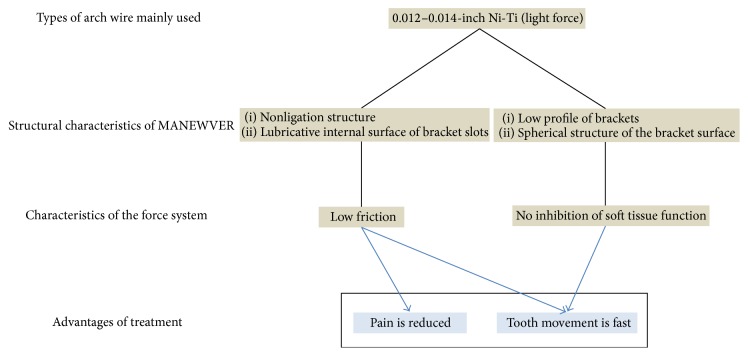
Properties of the new orthodontic treatment system.

To reposition teeth that have deviated from their normal position, it is considered that the new orthodontic treatment system can achieve a faster tooth movement rate than conventional methods (Figure 8). It is believed that this is because (1) a new zirconia bracket with multiple slots is nonligated, (2) the internal surface of the slots is lubricative because the bracket is produced by the zirconia firing working method, and (3) friction occurring in the wires is minimized by using 0.012−0.014-inch Ni-Ti arch wires. Furthermore, the involvement of load force applied by soft tissue in the dentition is estimated. Although it was clarified by Frederick [10] that the muscles surrounding the oral cavity and tongue have an important relationship with the position of the teeth, because the bracket surface structure of a new zirconia bracket with multiple slots is spherical, very small, and thin, it is unlikely that it hinders their function. Furthermore, Blake and Bibby [11] reported that the expansion of the mandibular intercanine width increases the risk of relapse. From the results of the present study, it is considered that because new zirconia bracket with multiple slots applies weaker forces than conventional methods, leveling is possible without expanding the mandibular intercanine width, and a more stable occlusal condition can be achieved. On the other hand, in cases requiring mesial movement of the molars and expansion, improvements in the movement period and efficiency are necessary.

Furthermore, in comparison with conventional orthodontic treatment methods, patient-reported pain did not readily occur as a result of using new zirconia bracket with multiple slots. It is considered that this was not only because the orthodontic force was low owing to the use of 0.012−0.014-inch Ni-Ti arch wires, but also because the friction occurring in the wire was minimal with new zirconia bracket with multiple slots.

In the future, it will be necessary to reconfirm the optimal load to move teeth in orthodontic treatment. This is important to prevent tooth root resorption and perform more effective tooth movement.

## 5. Conclusion

The purpose of the present study was to clarify the properties of new zirconia bracket with multiple slots regarding the tooth movement rate, changes in the morphology of the dental arch at the early stage of leveling, and pain caused by orthodontic treatment, by comparing the new orthodontic treatment system with a conventional treatment system. The proposed new orthodontic treatment system showed a higher tooth movement rate in the early stage of leveling, and the mean treatment period until the completion of leveling was significantly shorter.

## Figures and Tables

**Figure 5:**
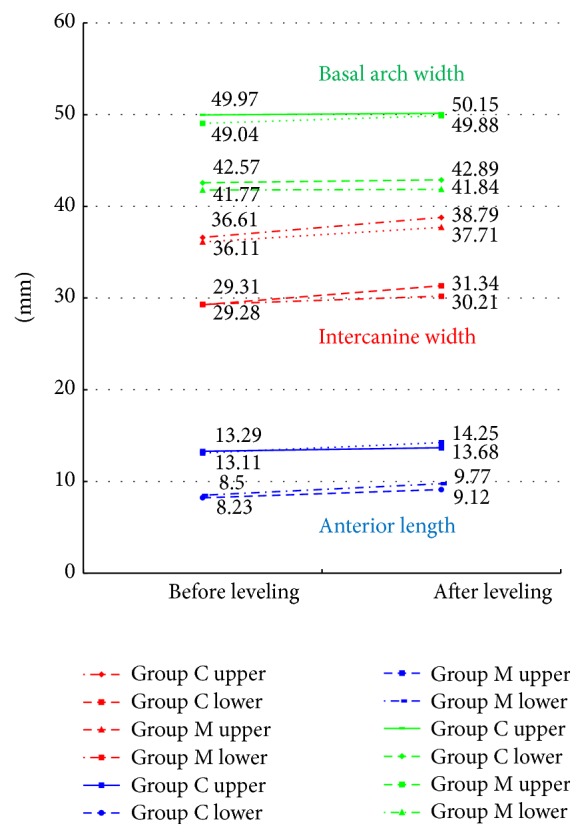
Changes in the dental arch morphology before and after leveling.

**Figure 7:**
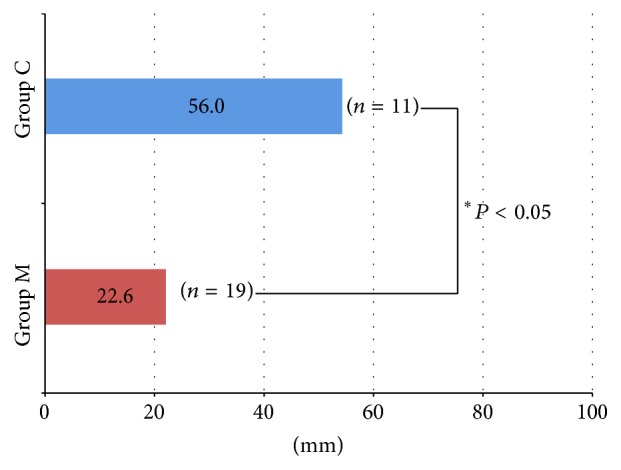
Pain caused by orthodontic treatment (visual analogue scale method).

**Table 1:** Mean treatment period until leveling completion.

		Days		
Group C	Upper	127.6	(*n* = 27)	*∗*
Group M	Upper	79.7	(*n* = 24)	

Group C	Lower	139.4	(*n* = 25)	*∗*
Group M	Lower	74.2	(*n* = 26)	

^*∗*^ *P* < 0.05.
